# Exudate compositions differ between the cover crops vetch and oat

**DOI:** 10.1038/s41598-026-44751-7

**Published:** 2026-03-22

**Authors:** Charlotte Turpin, Caroline Mauve, Anaïs Rattier, Bertrand Gakière, Annick Basset, Nathalie Harzic, Marie Garmier, Pascal Ratet

**Affiliations:** 1 Institute of Plant Sciences Paris-Saclay, Université Paris-Saclay, CNRS, INRAE, Université Evry Paris-Saclay, Gif sur Yvette, 91190 France; 2 Institute of Plant Sciences Paris- Saclay, Université Paris Cité, CNRS, INRAE, Gif sur Yvette, 91190 France; 3 Cérience, 1 allée de la sapinière, la Litière, Saint Sauvant, 86600 France

**Keywords:** Soil carbon, Rhizodeposition, Roots, Exudates, Primary and specialized metabolites, Ecology, Ecology, Microbiology, Plant sciences

## Abstract

Enhancing carbon (C) storage in soils through crop management can mitigate atmospheric CO₂ levels and improve soil health. Cover crops like bristle oat (*Avena strigosa*) and common vetch (*Vicia sativa*) may contribute to soil C sequestration via root biomass and rhizodeposition, including root exudates, but C storage potential at the species and varietal levels for these plants remain poorly documented, partly due to difficulties in measuring root exudation under field conditions. This study compared four cultivated varieties of bristle oat and common vetch, focusing on their root and exudate C content and metabolic profiles. Distinct metabolite profiles were identified mainly at the species levels. Oat roots had a reduced C content compared to vetch roots although they contained more amino acids, sugars, organic acids and specialized metabolites. On the contrary, C content was higher in oat exudates with more fatty acids and specialized metabolites while vetch exuded more sugars, organic acids and nucleotides. These results suggest that the mixture of these two plants can produce complementary C deposits, enhancing C storage and allowing the construction of a rich (microbial) soil ecosystem.

**Supplementary Information:**

The online version contains supplementary material available at 10.1038/s41598-026-44751-7.

## Introduction

Agriculture faces multiple challenges in terms of primary production, ecosystem services, resource conservation, and adaptation to climate change. The storage of carbon (C) in soils through agricultural practices can help limit the increase in atmospheric CO_2_ concentration, which is partly responsible for rising global temperatures^1^. This reduction can be achieved through direct C sequestration by crop plants. Additionally, the aboveground and belowground part of plant can prevent soil degradation by soil coverage and increase its fertility through the incorporation of plant material into the soil, through rhizodeposits^2,3^. Rhizodeposits include organic material derived from the microbial decomposition of plant litter, the release of border cells from the root cap and the production of exudates by roots and border cells^4–6^. This soil improvement and C storage can be achieved through better crop management^7^, which can in addition help reduce the use of agricultural inputs (nutrients and pesticides^8)^. Improved soil fertility can enhance crop robustness, water retention in the soil, and the restoration of soil microbial biodiversity^9^, which has a beneficial effect on controlling the proliferation of soil-borne pathogens^10^.

These improvements can be obtained through agroecological practices^11^ or agricultural management practices like increased used of cover crop cultures^12^. Cover crops are the non-cash crops grown between the harvest and the next planting of main crops^13^. The use of cover crops for reducing bare surfaces during winter is a major lever for increasing soil C storage^14–16^. However, the capacity of plants to store C in the soil is poorly documented at the interspecific level, and the literature lacks data to characterize cover crops at the varietal level^17,18^. This lack of data is partly linked to the difficulty of quantifying in situ the root biomass produced as well as root exudation, which constitutes an essential part of rhizodeposition^19,20^. In addition, studies to understand the role of cover crops as C sequesters are less developed, despite the fact that they may represent interesting tools for C soil storage^21–23^ and microbiome shaping^16,18,24–26^.

Carbon deposition in soil by (cover) crops originates from photosynthesis and can come from the root or aboveground part of the plant introduced into the soil at the end of the culture^2,20,27^. Root exudation is also responsible for nutrient release in soils. Plant roots exude primary metabolites, including organic acids, sugars, amino acids, nucleotides, and specialized (or secondary) metabolites^6,28,29^. They also produce high-molecular weight compounds like mucilage and proteins. Specialized metabolites have been studied in detail because of their possible pharmaceutical interests and also as they potentially act as signaling molecules in the rhizosphere^30,31^. Primary metabolite exudation is less documented, but it is believed that large amounts of the photosynthate products released in the rhizosphere are derived from primary metabolites and can shape the microbiome to the benefit of the plant^24,30,32,33^. Studying exudates is challenging because culture methods used in most studies may not reproduce field conditions. Most studies are done in axenic conditions in vitro, which are very different from the soil and microbiome environment of plant roots in the field. Some studies have addressed this issue^34,35^ but have questioned the wounding effect on the root when taking them out of the soil and the identification of a limited number of compounds, some of which can also be attributed to the microbiome. Nevertheless, the studies by^34,35^ indicated that the major classes of metabolites can be identified using hydroponic versus soil growing conditions.

Plant mixtures, including cereal and legume plants, can have a beneficial effect on C and nitrogen (N) sequestration in soil and consequently on the establishment of a beneficial microbiome^18,36,37^. The choice of these mixtures is often made according to criteria of above-ground biomass production and adaptation to cropping systems^17,38^. The root architecture that can structure the soil is also a criterion for plant mixture composition. Previous studies on plant exudates were done using single plants like crops, but as cover crops are often used as mixtures, it is of interest to describe their exudate production in terms of complementarity^18,39^.

Among the diverse species used in cover crop mixtures, bristle oat and common vetch are largely adopted due to their complementary ecological functions and adaptability to various cropping systems^40^. Bristle oat is an annual grass species known for its rapid growth, high biomass production, and soil-structurating properties due to its root architecture^41,42^. It develops a fibrous, dense root system, with many fine roots spreading widely and penetrating soil layers, enhancing water and nutrient uptakes. Bristle oat is used as cover crop in fall and winter or, being highly palatable to herbivores, as a spring and summer forage crop. Common vetch is a leguminous species with root development characterized by a slender branched taproot^41,42^, valued for its symbiotic nitrogen-fixing capacity with rhizobia and soil fertility enhancement^43^. In addition to the complementary ecological functions of these two cover crop plants, we aimed to describe their role in soil C deposition. For this, we compared the root and exudate C profile of four different cultivated varieties of bristle oat (*Avena strigosa*) and common vetch (*Vicia sativa*) and investigated if primary metabolites differ between the two species.

## Results

### Experimental setup and plant growth characteristics

**Fig. 1:**
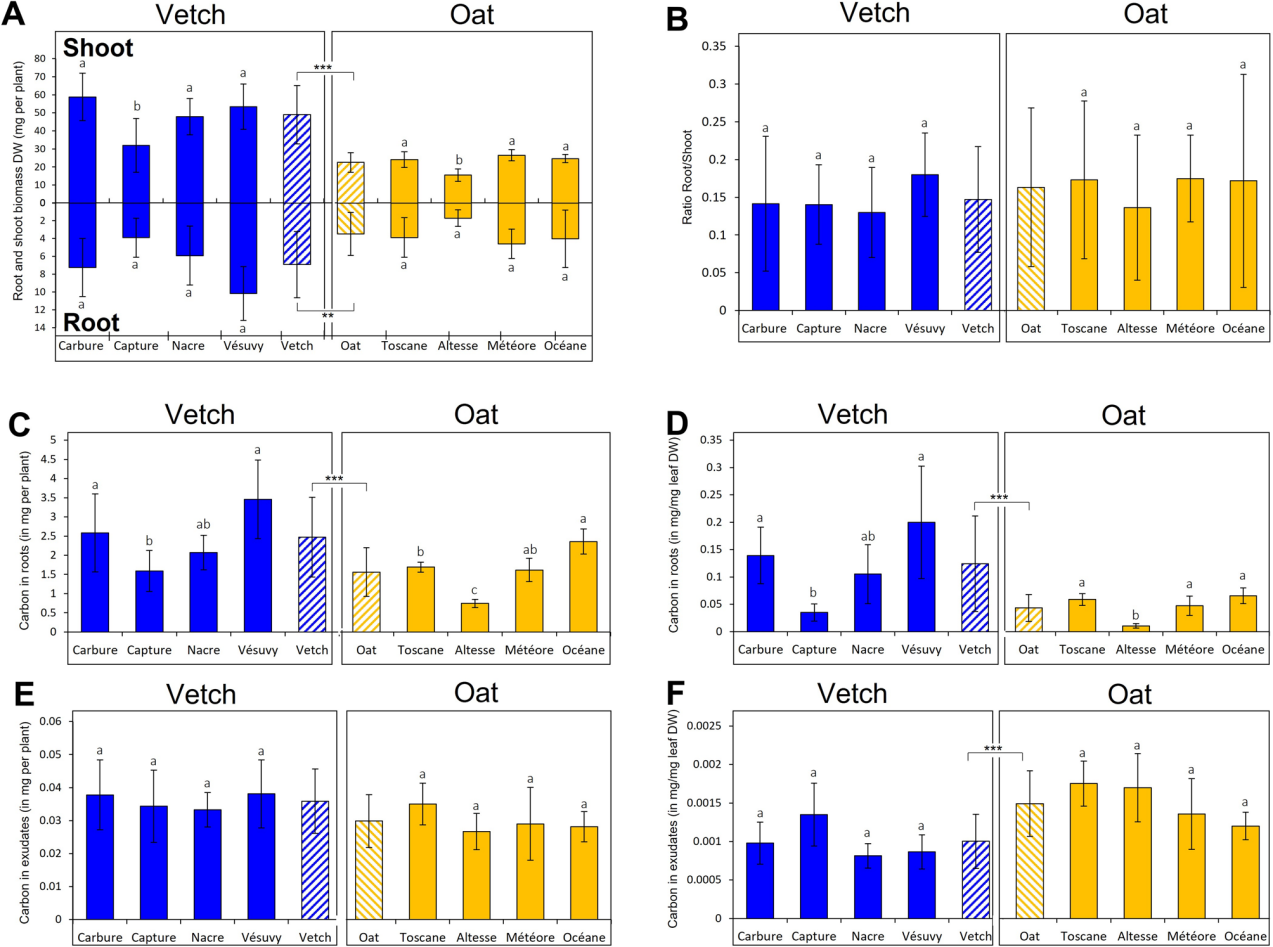
Plant biomasses and carbon contents of roots and exudates for vetch and oat varieties. (**A**) Shoot biomass (upper part of panel A) and root biomass (lower part of panel A) in mg of dry weight (DW) per plant for vetch (dark blue) and oat (orange) varieties. The hatched histograms correspond to the average biomasses of the different varieties of vetch or oat. (**B**) Ratio root/shoot biomasses. The ANOVA analysis indicates no significant difference. (**C**–**F**) Carbon contents in roots (C, D) and root exudates (E, F) in mg per plant (C, D) or mg/mg leaf DW (E, F) for the different varieties. The hatched histograms correspond to the average C contents in vetch and oat. From A to F, error bars represent standard deviations. Different letters (a, b) indicate a significant difference between vetch and oat varieties (*p* = 0.05). Significant differences between vetch and oat species are indicated over and below panel A and in panel C, E and F (*** *p* < 0.001; ** *p* < 0.01). For each variety, *n* = 64 for leaves and *n* = 32 to 48 for roots in panel (A) For each variety *n* = 48 in panel (B) For each variety *n* = 10 (1 sample is a pool of 8 plants) in panels C to F.

Four varieties of the common vetch *Vicia sativa* L. (Carbure, Capture, Nacre, and Vésuvy) and four varieties of the bristle oat *Avena strigosa* Schreb. (Toscane, Altesse, Météore, and Océane) (hereafter referred to as vetch and oat) were grown in hydroponics, a culture system that allows easy collection of roots, root exudates, and metabolic profiling analyses (Supplemental Fig. S1). Exudate, root, and shoot materials were collected after 10 days of hydroponic growth. At that time, the average dry biomass (shoot and root biomasses) of vetch was higher compared to oat (Fig. 1A). Capture and Altesse had reduced dried shoot biomasses compared to other varieties of vetch and oat, respectively. However, their root biomasses (Fig. 1A) and shoot/root biomass ratios (Fig. 1B) were not statistically different. Thus, the varieties exhibited homogeneous growth except for Capture and Altesse, which may have a reduced size or a slower growth.

### Carbon content of roots and exudates

The average C content in roots per plant or per mg of leaf dry weight (DW) was significantly higher for vetch compared to oat (2.42 mg C per plant for vetch and 1.60 mg C per plant for oat, and 0.12 mg C/mg DW leaf for vetch and 0.05 mgC / mg DW leaf for oat; Fig. 1C and D). Capture (vetch) and Altesse (oat) varieties had the lowest values for root C content.

In contrast, oat had significantly more C in exudates than vetch when expressed in DW of leaf material (0.001 mg C/mg leaf DW for vetch and 0.0015 mg C/mg leaf DW for oat) although there was no difference between vetch and oat C content when expressed per plant (Fig. 1E and F). There was no significant difference in the C content of the exudates between the four varieties of the two plant species. Thus, although vetch produced more shoot and root biomasses, with more C in roots, it did not exudate more C from its roots compared to oat. On average, oat seemed to exude more C but only when data were reported relative to the leaf biomass, suggesting an increase in the flux of C from leaf to the outside of the roots. The total C content per plant of the root system was on average 2.42 mg C per plant for vetch and 1.60 mg C per plant for oat. In exudates, the C content per plant is 0.035 mg C per plant for vetch and 0.029 mg C per plant for oat. This indicates that in this experimental setup, there is 55 to 70 times more C in the root system than the C exuded in 24 h. In other words, the root system can exude 1.44% for vetch and 1.81% for oat of its root C content equivalent in 24 h.

### Non-targeted metabolite profiling of roots and root exudates

**Fig. 2:**
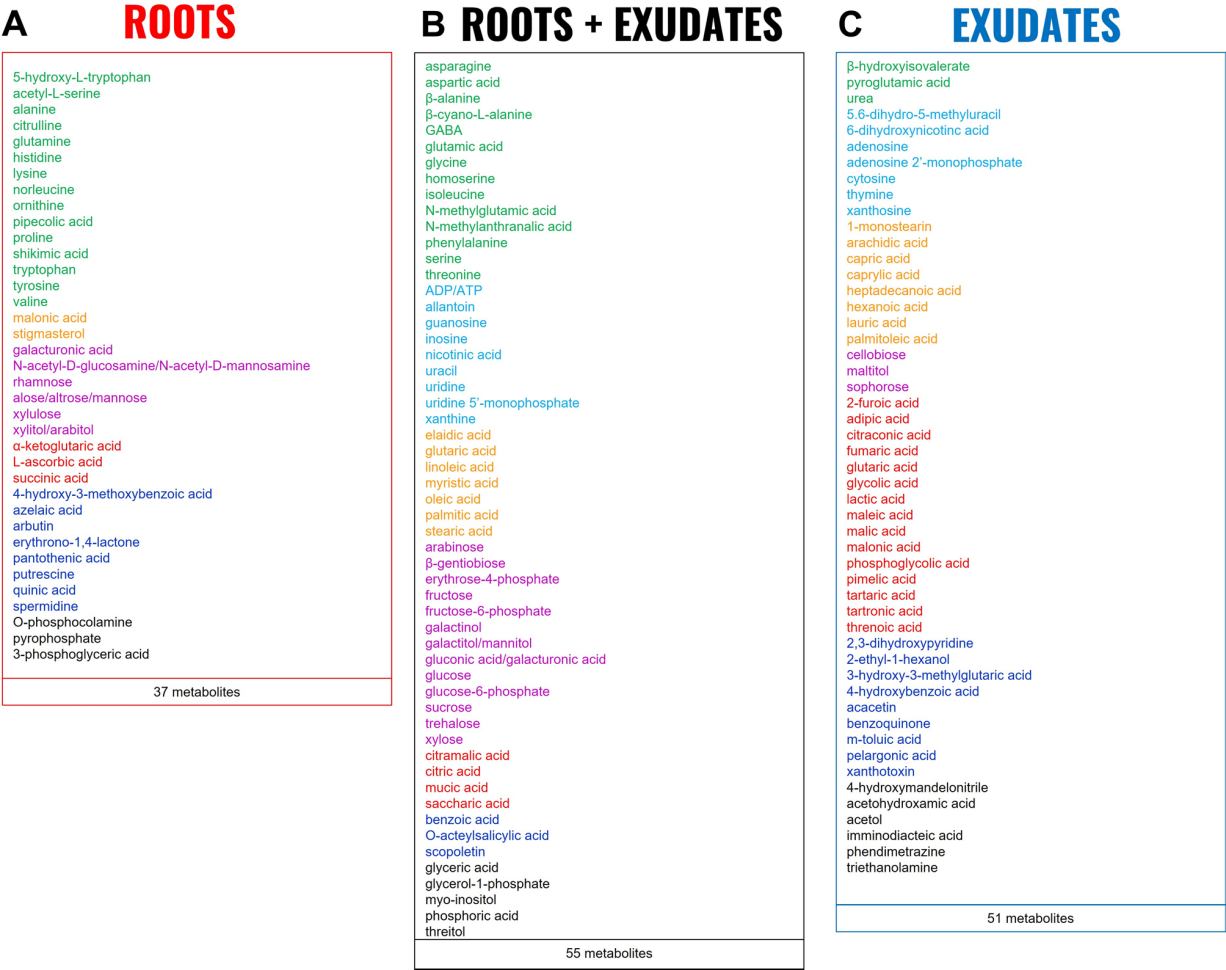
Metabolites identified in roots and root exudates of oat and vetch by GC-MS. Metabolites identified in roots (**A**: 37 metabolites), in roots and exudates (**B**: 55 metabolites) and in exudates (**C**: 51 metabolites) in vetch and oat. Metabolites are further colored by class: green: amino acids and derivatives; light blue: nucleic acids and derivatives; orange: fatty acids and lipidic compounds; pink: sugars, sugar alcohols and derivatives; red: organic acids; dark blue: specialized metabolites; black: others. The names of metabolites separated by a / correspond to isomers or molecules with similar chemical structures that could not be differentiated by GC-MS. *n* = 18 for vetch (note that the root samples for the Nacre variety are missing in this analysis but exudates have been analysed for all varieties) and *n* = 20 for oat, with a pool of 8 plants for each sample.

Using a non-targeted Gas Chromatography - Mass Spectrophotometry (GC-MS) analysis, a total of 143 metabolites were identified in roots and exudates of the two plant species, including amino acids, nucleic acids, fatty acids, sugars, organic acids, specialized metabolites, and other metabolites (Fig. 2 and Supplementary Table 1). These metabolites were classified into three groups: metabolites only identified in roots (37 metabolites, 26%), metabolites only identified in exudates (51 metabolites, 36%), and metabolites identified in both compartments (55 metabolites, 38%) (Fig. 2). This represents 92 metabolites identified in roots and 106 in exudates. Importantly, all the metabolites identified in roots were found in both plant species (Table 1).

Among the 143 metabolites identified in the two plant species, 22% were amino acids, 12% nucleic acids, 12% fatty acids, 17% sugars, 16% organic acids, 12% specialized metabolites, and 9% other metabolites (Supplementary Table 1). Each class of metabolites is differently represented in the three groups (roots, exudates, and both), with an over-representation of amino acids and sugars in roots and under-representation in exudates. Organic acids are over-represented in exudates and under-represented in roots. Specialized metabolites are over represented in specific root and exudate metabolites, meaning that few are common to both compartments. In addition, nucleotides are absent from the root-specific metabolites. There was no root metabolite specific for one plant species as all root metabolites were identified in the two plant species, but four metabolites were identified only in oat exudates (fatty acids) and seven in vetch exudates (including 5 nucleic acids) (Table 1), suggesting a metabolite signature of the two species.

**Table 1:** Specific metabolites in vetch and oat roots and exudates. Bold: nucleic acids and derivatives; italic: fatty acids and derivatives; bold italic: sugar and underline: organic acids.

Roots		Exudates	
Vetch	Oat	Vetch	Oat
No metabolites detected in only 1 species		**guanosine**	*1-monostearin*
		**inosine**	*arachidic acid*
		**uridine**	*caprylic acid*
		**uridine-5′-monophosphate**	*linoleic acid*
		**xanthosine**	
		***glucose-6-phosphate***	
		mucic acid	

**Fig. 3:**
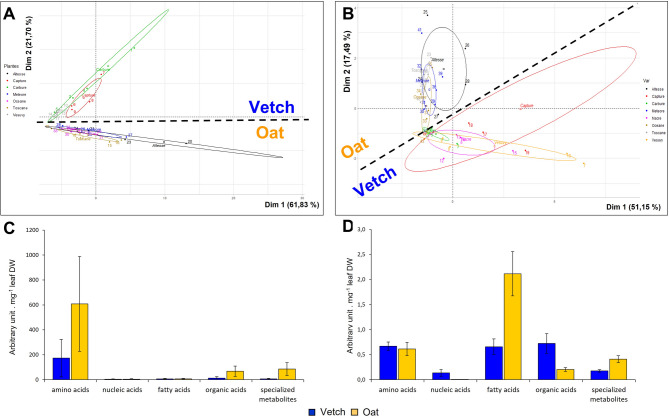
Metabolic profiles of roots and exudates of oat and vetch. (**A**, **B**) Principal component analysis (PCA) of 92 metabolites in roots (A) and 106 metabolites in exudates (B) in vetch (3 varieties for the root samples, the Nacre variety is missing; 4 varieties for the exudates) and oat (4 varieties). (**C**, **D**) Sum of metabolite content (in arbitrary GC-MS unit/mg leaf DW) for each class of metabolites for both plant species (C for roots and D for exudates). Sugar values are not shown here (see below). Error bars represent the standard deviation of the sum of the metabolite content of each variety.

Next, we wanted to compare in more detail the presence and amount of each identified metabolite in the two plant species and in their varieties, in roots and in exudates. Principal Component Analysis (PCA) of the metabolites detected in roots (Fig. 3A) and exudates (Fig. 3B) clearly distinguishes between vetch and oat species, highlighting their distinct metabolic profiles. The same analysis performed within vetch or oat varieties did not allow differentiation between these varieties, suggesting that there was no significant difference in metabolite profile and content between them (Fig. 3A and B). In agreement with this PCA analysis, the root and exudate metabolic profiles for vetch and oat are distinct when looking at the quantity of the different classes of metabolites (Fig. 3C and D) with more abundance of total amino acids, organic acids and specialized metabolites in oat roots and more abundance of total fatty acids in oat and organic acids in vetch exudates. Sugar values are not shown in Fig. 3 because they were very abundant and were studied separately (see below). This suggests that sugars represent a large amount of the C containing compounds for both roots and exudates for the two plant species.

**Fig. 4:**
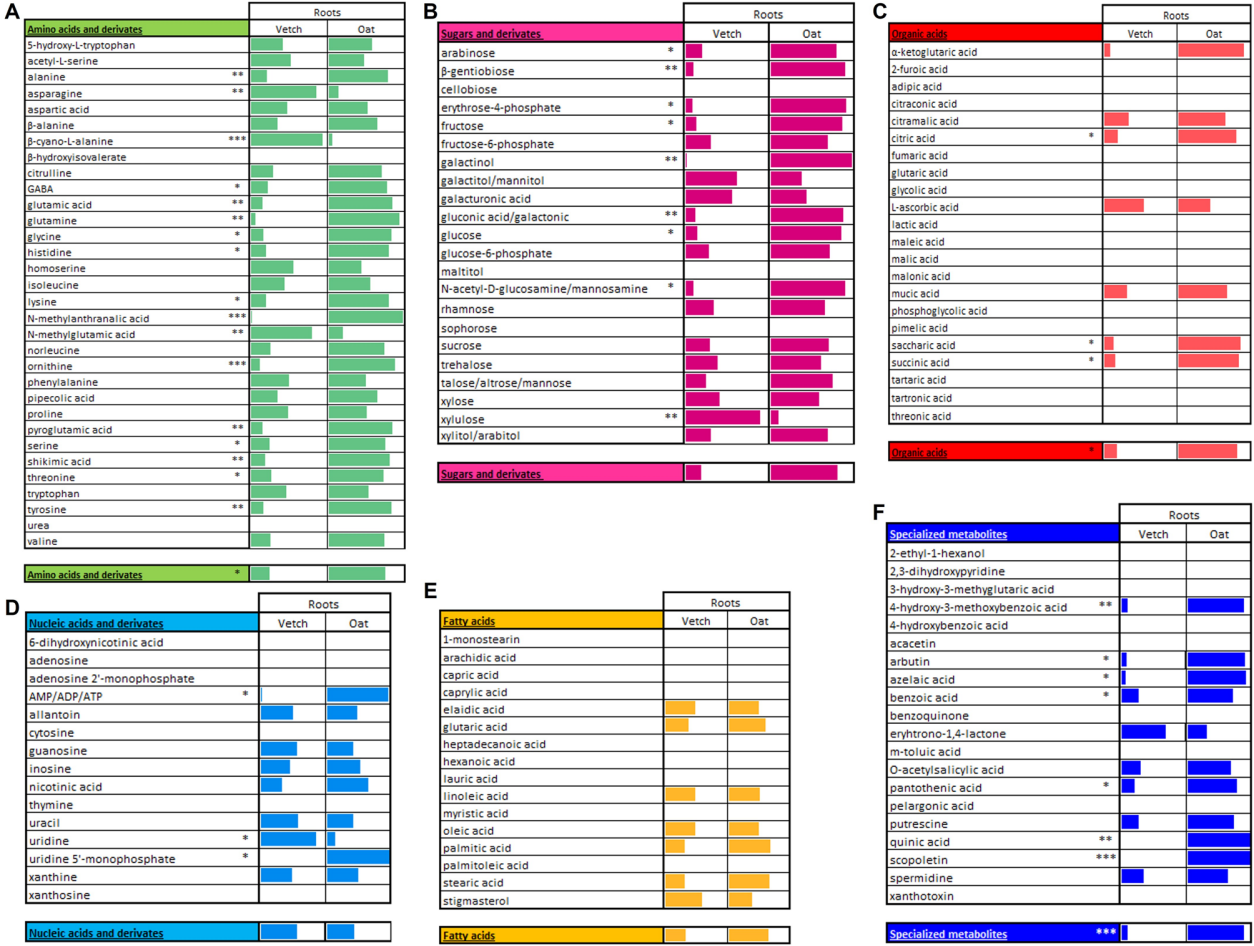
Gauge representation used to compare absence/presence and relative proportions of metabolites in roots of oat and vetch. (**A**) amino acids and derivatives, (**B**) sugars, sugar alcohols and derivatives, (**C**) organic acids, (**D**) nucleic acids and derivatives, (**E**) fatty acids and lipidic compounds and (**F**) specialized metabolites. For a given metabolite, the length of the colored rectangle represents the percentage of relative quantity in a species (the sum for the two species is 100%). The longer the colored rectangle is in one species for one metabolite, the more it accumulates compared to the other species. It is not possible to compare the proportions between different metabolites with this data representation. The single line below each class of compound represents the average percentage of each metabolite. Significant differences between vetch and oat are indicated as: * *p* < 0.05; ** *p* < 0.01; *** *p* < 0.001. *n* = 4–5 for each variety.

**Fig. 5:**
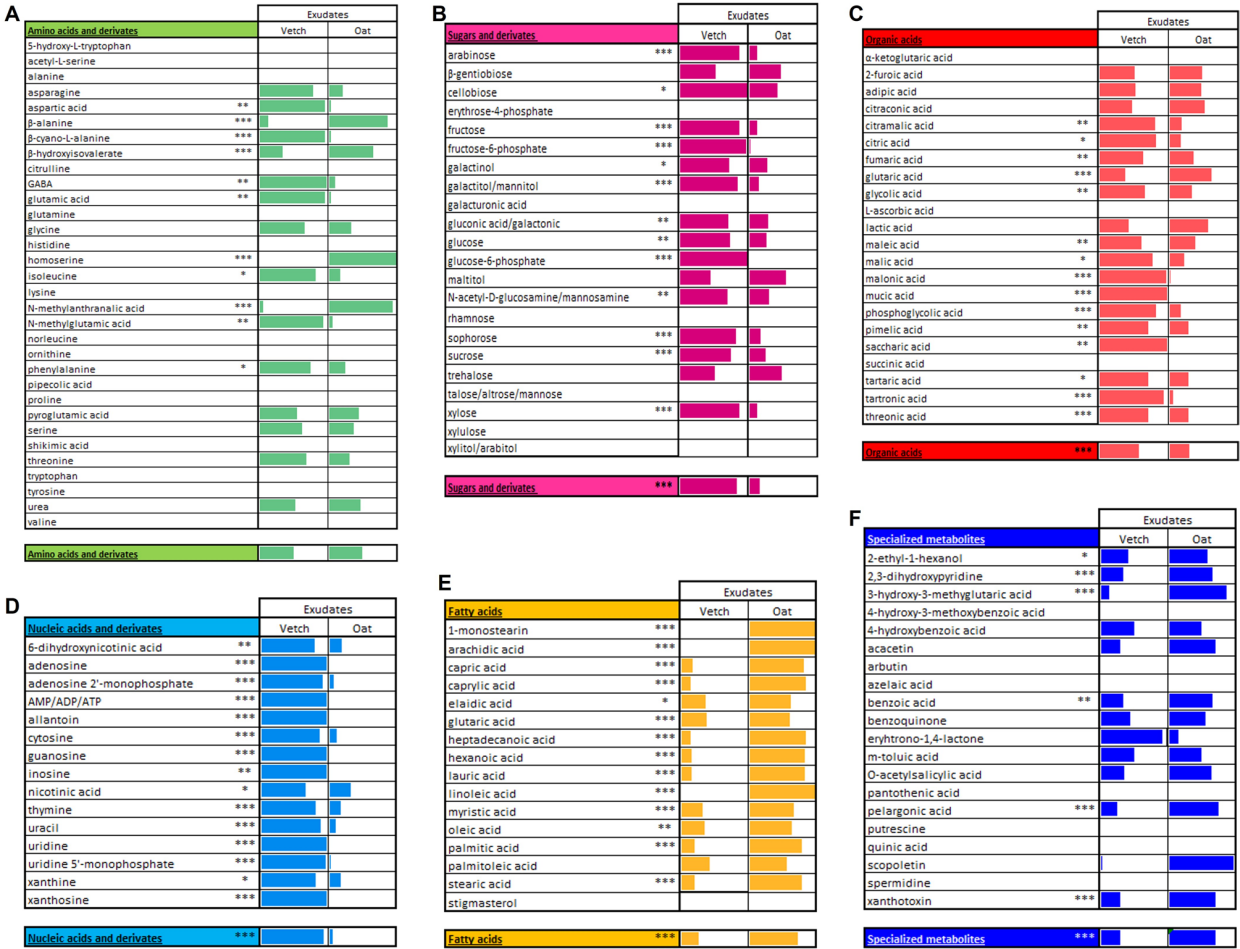
Gauge representation used to compare absence/presence and relative proportions of metabolites in exudates of oat and vetch. (**A**) amino acids and derivatives, (**B**) sugars, sugar alcohols and derivatives, (**C**) organic acids, (**D**) nucleic acids and derivatives, (**E**) fatty acids and lipidic compounds and (**F**) specialized metabolites. For a given metabolite, the length of the coloured rectangle represents the percentage of relative quantity in a species (the sum for the two species is 100%). The longer the colored rectangle is in one species for one metabolite, the more it accumulates compared to the other species. It is not possible to compare the proportions between different metabolites with this data representation. The single line below each class of compound represents the average percentage of each metabolite. Significant differences between vetch and oat are indicated as: * *p* < 0.05; ** *p* < 0.01; *** *p* < 0.001. *n* = 4–5 for each variety.

Because the absolute quantification in GC-MS analysis is time consuming, we did only relative quantification to compare the amounts in arbitrary units for each compound in the same compartment. For this comparison, the estimated relative amount in percentage of each metabolite is represented using gauges comparing the presence and relative amount of one metabolite in the two plant species. The results for the roots are presented in Fig. 4, and the results for the exudates in Fig. 5. This comparison was done only between the species because the varieties behave similarly within one species. Using this representation, one can compare the relative amount of each metabolite in the two species for either roots or exudates. For example, adenosine 5’-triphosphate or diphosphate (ATP or ADP) accumulates at higher levels in oat roots than vetch roots (Fig. 4D). However, in the exudates, ATP or ADP is detected in vetch but nearly absent in oat (Fig. 5D).

A similar diversity of compounds was observed within each class of compounds in the two plant roots and exudates. However, the amount for each class was different in each species. For example, there were more amino acids, sugars, organic acids and specialized metabolites in oat roots, but more nucleotides in vetch roots (Fig. 4). Interestingly, significant differences were observed for sugars like glucose and fructose, which were more abundant in oat roots (see also next chapter). In exudates, sugars, organic acids and nucleic acids including guanosine, inosine, uridine, uridine 5’-monophosphate and xanthosine were more abundant in vetch exudates. In contrast, fatty acids, including arachidic acid, linoleic acid, oleic acid, and palmitic acid as well as specialized metabolites were more abundant in oat exudates (Fig. 5).

In conclusion, this analysis highlights the different patterns of metabolites found in the exudates of these two plant species, which could potentially complement each other in cover crop mixtures. At least for these metabolites, there was no difference in metabolic diversity and relative amount between the varieties studied for each species.

### Sugar quantification in roots and exudates

The GC-MS analysis indicated that sugars represent a large amount of the C-containing compounds for both roots and exudates for the two plant species. This analysis also indicates that oat roots contain more sugars, including sucrose, glucose, and fructose, than vetch roots and, on the contrary, vetch exudates contain more sugars than oat exudates. These three sugars had the strongest signal (highest arbitrary GC-MS values) observed in the GC-MS analysis. To confirm these differences, we measured their concentrations using quantitative methods: an enzymatic assay for sugar quantification in roots and High-Performance Liquid Chromatography (HPLC) quantification for the exudates (see Methods).

**Fig. 6:**
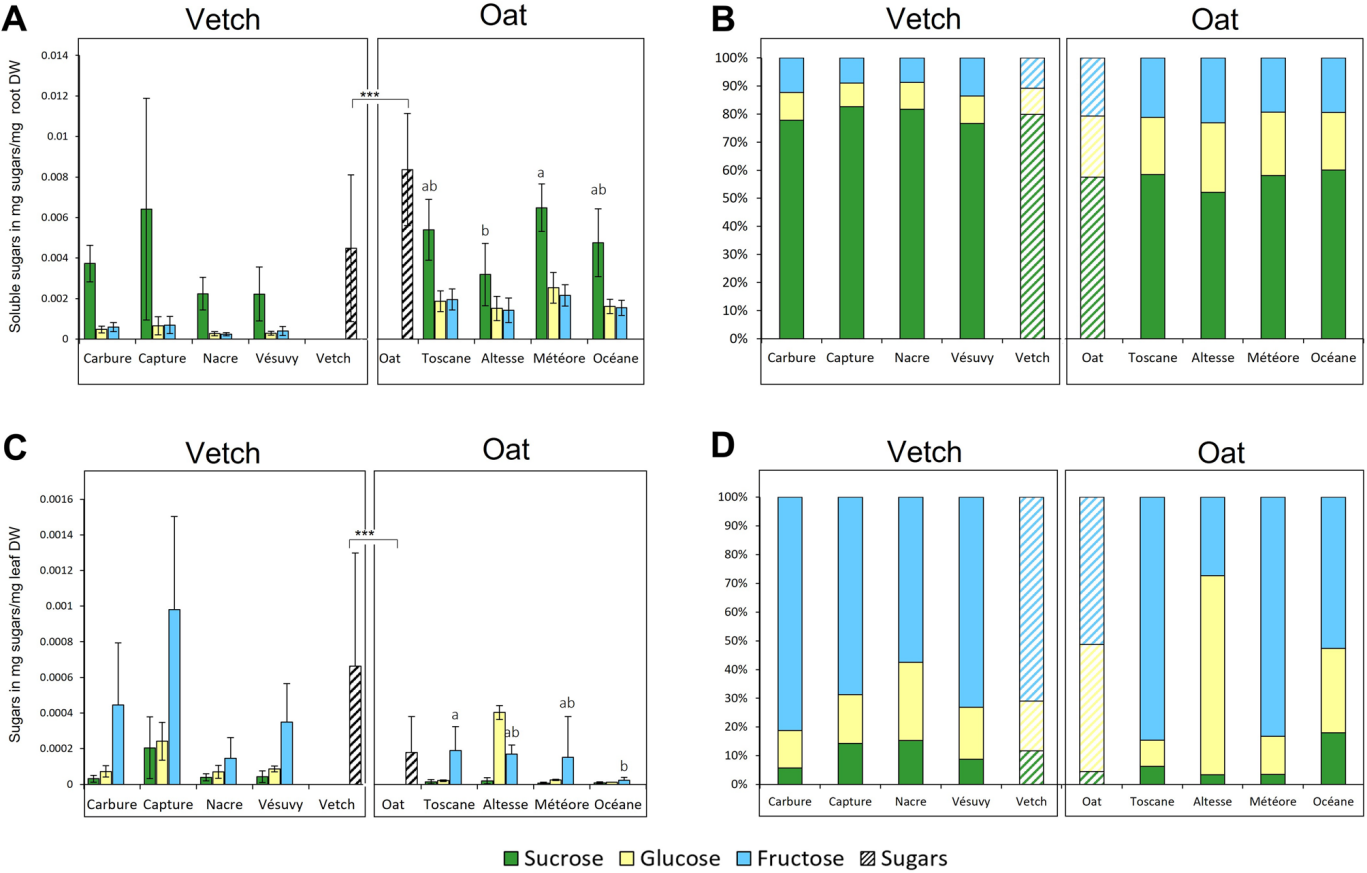
Sugar quantification in roots and root exudates of oat and vetch varieties. (**A**, **B**), quantification of soluble sugars (sucrose, glucose and fructose; in mg sugars/mg root DW) in roots using an enzymatic assay (A). Proportion of the three sugars in roots (B). (**C**, **D**), quantification of soluble sugars (sucrose, glucose and fructose; in mg sugars /mg leaf DW) in exudates using HPLC analysis (C). Proportion of the three sugars in root exudates (D). In A to C, the hatched histograms correspond to the average values of total sucrose, glucose and fructose amounts in vetch and oat (average made on the different varieties). Error bars represent standard deviations. Different letters (a, b) indicate a significant difference (*p* = 0.05) between varieties. Significant differences between vetch and oat species are indicated as *** *p* < 0.001. *n* = 8 for each variety in A and B. In C and D *n* = 2–5 (for some sugars and samples, data were removed as they were below the detection threshold).

The enzymatic assay confirms that oat roots contain more sugars (sucrose, glucose, and fructose) than vetch roots (Fig. 6A). There was no significant difference in sugar content and composition between the vetch varieties. Still, in oat, the sucrose and total amount of sugars were significantly lower for the Altesse variety compared to other oat varieties (Fig. 6A). Additionally, the proportion of sugars in roots differs depending on the species, but sucrose was the most abundant sugar for both plants. In vetch roots, the respective proportions are ~ 80% for sucrose, ~ 9% for glucose, and ~ 11% for fructose. In oat roots, the respective proportions are ~ 55.5% for sucrose, ~ 22.5% for glucose, and ~ 22% for fructose (Fig. 6B).

Because the enzymatic assay was not sensitive enough to measure the amount of the different sugars in exudates, we measured them using HPLC. However, even with this more sensitive method, sugars like sucrose and glucose were present in amounts at the limit of detection in oat exudates. Our analysis shows that vetch varieties have more sugars in exudates compared to the oat varieties (Fig. 6C), in agreement with the GC-MS analysis. We did not observe significant differences in sugar composition between the vetch varieties. In contrast, in oat, Altesse might have more glucose in exudates than the other oat varieties. Interestingly, as observed in roots, the proportion of the three sugars in exudates differed between the two species (Fig. 6D). However, in contrast to roots, fructose was the most abundant sugar in the exudates of both species. In vetch, fructose represents ~ 60% of the three sugars, glucose ~ 18%, and sucrose ~ 12%. In oat, fructose represents ~ 54% of the three sugars, glucose ~ 37%, and sucrose 9%. This indicates that the sugar composition observed in exudates does not directly reflect the sugar composition of the roots, and this composition in both compartments is different between the two species.

## Discussion

This study aimed to evaluate C content of cover crop roots and root exudates as well as their metabolic composition. Cover crops provide several ecological services, including soil protection between two crops^12,16^. They can also participate in C sequestration through the exudation of different primary and specialized metabolites, the incorporation of plant root and aboveground biomass after the cover crop culture or by shaping the microbial communities. The direct measurement of C deposition by crops in soil is possible^4^, but the identification of the compounds released by the plant in the rhizosphere is still challenging^35^. In this work, we chose to use a hydroponic system to evaluate and compare root and exudate C contents of both *Vicia sativa* (common vetch) and *Avena strigosa* (bristle oat). Plants grown under hydroponic conditions produce exudates with different compositions than plants grown in non-sterile soils, but the classes of compounds produced (sugars, amino acids, etc.) are similar^34,35^. This hydroponics system facilitates root and exudate collection and analysis and allows the identification of a larger number of metabolites and avoids analyzing those produced by the microbiome of the rhizosphere^31,39^. For this study, several tests were carried out to determine the best parameters for exudation and metabolite collection/identification: plant growth stage, exudation time, volume and medium. Exudation with younger plants (less than 10 days in hydroponics) did not allow quantification of sugars in the exudates or required a longer exudation time. The 24 h exudation time in water was chosen because it allowed the best qualitative and quantitative detection of metabolites by chromatography. This longer exudation time maximized the number and intensity of compounds detected as shown by^39^.

Oat roots had a reduced C content compared to vetch roots even when calculated by mg of dry leaf material indicating that the root C allocation is species dependent. One goal of this study was also to highlight possible differences in C content and compositions between the four varieties within each species. However, our analysis revealed few differences between the varieties studied here, except for Capture in vetch roots and Altesse in oat roots. This result might indicate that potential differences between varieties may not have been large enough to be detected by the techniques used in this study.

The C content of the exudates of the two plant species, calculated per plant, were similar. In contrast, when calculated per mg of dry leaf material, the exudated C content of oat was higher, suggesting a greater allocation of photosynthates from the aboveground parts to the root exudates in oat. It could result from the presence of fatty acids with long carbon chains (like arachidic acid, heptadecanoic acid, or 1-monostearin) in these oat exudates. The root C content per plant (in mg of C per plant) was 50 to 70 times higher compared to the exudate C content, even if exudates were collected for only 24 h and the roots were 13 days old. Calculating per day of plant development, this corresponded to a C allocation of 80% to the root and 20% to the exudate, in agreement with^32^. This suggests that C storage in soil by these species may depend more on the root biomass and eventually the aboveground part produced in the field^44^ rather than the amount of C released by the plant in the rhizosphere in the form of exudates. However, previous studies indicate that the long-term effect on soil C stocks varies between the C sources (exudates, roots, and aboveground biomass) and that exudates and rhizodeposited C are relatively more important for soil C buildup^3,27^, adding complexity to the interpretation of quantitative comparisons between soil C input pools^45^.

To have a more complete view of the root and exudate C compositions, we decided to complement the C measurement with a more detailed analysis focused on primary metabolite production using a combination of techniques, including HPLC and GC-MS, which allow detecting and/or quantifying sugars, amino acids, and other metabolites including some specialized metabolites. GC-MS is a sensitive and reliable technology with highly reproducible mass spectra and extensive developed spectral libraries, allowing both qualitative identification and quantification of metabolites^46^. It is particularly suitable to study primary metabolites that are dominant among root exudates of plants^47,48^. HPLC and enzymatic assays were used as complementary techniques to accurately quantify the free sucrose, fructose, and glucose contents in root and exudate samples.

The metabolites identified in the roots of the two species in our study were the same, even if the amount of each of them can vary, with, for example, more amino acids and derivatives in oat roots. This result is different from those described in the study by^39^, but might be the consequence of the different plants used in the two studies but also of the different media and culture conditions. In addition, the analysis method is different. In our study we also found that oat roots had more free sugars, organic acids and specialized metabolites. The higher content in free sugars in oat roots compared to vetch roots is surprising because they have a lower C content. The presence of other compounds like specialized metabolites and cell wall constituents in vetch might explain these differences. Interestingly, exudates of the two plant species can be differentiated by their composition, in agreement with the study of^39^, showing common and specific compositions of root exudates of Arabidopsis, Medicago, and Brachypodium plants and suggesting the specific exudation of metabolites by roots^32^. Vetch exuded more free sugars, organic acids and nucleotides, while oat was characterized by more fatty acids and specialized metabolites in its exudates. Fatty acids were not described (or detected) in^39^, but were clearly differentiating the two species in our analysis and may represent a specific signature for oat exudates. Lipids exuded by plants play different roles in the rhizosphere including interaction with the microbiome^49^ as well as participating in the C storage in soil^50^. It is known that legume exudates are characterized by their richness in flavonoids and derivatives as specialized metabolites^31,37,51^, but molecules like flavonoids were not detected using our technology. Our study additionally highlighted a higher sugar content and a specific production of nucleotides in vetch exudates. In the study by Liu et al.^52^ nucleotides present in exudates were correlated to the recruitment of a specific microbiome during heat stress. These analyses may suggest that, in addition to specialized metabolites playing a role in plant-microbe communication^25,30^, primary metabolite diversity between species may also play a role in plants to shape their biotic environment. This is also the case of the amino acid threonine detected in the exudate of the two plants studied here that was shown by^53^ to stimulate the phosphorus solubilizing activity of bacteria. This might point to a more specific role of exudates, in addition to C deposition, in structuring the microbiome as a consequence of their specific composition^26,54,55^. Our study is however a first step in the description of the exudate composition, because we analyzed only exudate composition of a single species at a time. We thus cannot exclude that growing two species together will not change the exudate composition of each of them, resulting in an exudate composition that will not be the addition of the two ones described in our study. An aim for future studies should thus be to analyze exudates when using these plants in mixture in laboratory conditions or in soil. However, our analysis is a starting point for such analysis. The absence of differences in metabolite composition between the varieties does not exclude that these differences may exist on metabolites not studied here like flavonoids for vetch or that the differences are quantitative but our methods were not sensitive enough to detect these differences. Such differences can be detected inside a species but may require more sophisticated technologies at the ecotype level^56^.

The choice of the exudation protocol in hydroponics conditions may have influenced the type and nature of compounds detected in this analysis as also noticed by^35,39,46,57^ and may not entirely reflect the exudate composition in the natural environment (soil in the field). In fields, the exudate composition may indeed depend on the plant age and nutrition, soil physical properties, plant-plant interactions^29^, microbiome composition and plant root architecture^35,39,46,58–61^. However, the authors of these studies^35,39,46,58–61^ also suggested that the same classes of compounds can be found in different quantities in soil. In non-sterile soil, the amount and number of metabolites detected are also reduced^49^. If indeed the same classes of metabolites described in our study are also produced in the field, the mixture of these two plants will produce complementary C deposits with a large panel of metabolite classes, allowing the construction of a rich ecosystem following these cover crop cultures. This may help establish positive microbial associations for the benefit of agriculture^36^. Investigating the root exudates and C content of different plant varieties and species could provide a promising avenue for selecting genotypes with enhanced agronomical performance and ecological resilience.

In conclusion, our work describes the root and exudate composition of four different commercial varieties of bristle oat (*Avena strigosa*) and common vetch (*Vicia sativa*) used as cover crops. Our results highlight significant differences in C content and (primary) metabolite composition of these plants, mostly at the species level. The differences were observed in most of the metabolite classes detected either in roots or in exudates. This work also showed that the hydroponic system used in our study represents a simple and reproducible set up to characterize and compare plant C deposition in future studies. By minimizing the effects of soil heterogeneity and microbial activity, this system is a useful tool to disentangle plant intrinsic controls on root exudation and to explore the biochemical diversity of plant-derived C compounds. It can help in future studies the mechanistic understanding of plant C allocation and belowground functioning. Furthermore, the generation of large and standardized datasets through root exudate analyses (‘exudomes’) will improve data comparability across studies and support integrative and meta-analytical approaches in root and rhizosphere research.

## Methods

### Plant material and growth conditions

Seeds of *Vicia sativa* L. (common vetch) and *Avena strigosa* Schreb. (bristle oat) were provided by the company Cérience (Beaufort en Anjou, F49250; https://www.cerience.fr/en). The varieties used were Carbure, Capture, Nacre and Vésuvy for common vetch and bristle oat varieties are named Toscane, Altesse, Météore and Océane. These varieties are registered in the French catalog of cultivated crops (https://www.geves.fr/catalogue-france/). For simplification, common vetch and bristle oat will be named vetch and oat in this article. Vetch seeds were surface sterilised with NaOCl (1.5 g chlorine/L) supplemented with a drop of washing-up liquid for 45 min with shaking and then rinsed 5 times with sterile water. Oat seeds were husked, then surface-sterilized with NaOCl supplemented with one droplet of dishwashing liquid for 20 min and then rinsed as for vetch.

Seeds were stratified on sterile water agar plates (15% agar) for two days at 4 °C in the dark and then allowed to germinate for 24 h at 24 °C in the dark. After germination, seeds were transferred to sterile Buffered Nodulation Medium (BNM) agar^62^(6 seeds/square plate) with roots sandwiched between two pieces of sterile paper for vertical growth under 16 h day at 24 °C and 150 µE and 8 h night at 19 °C for 3 days. Plantlets of homogeneous size were then transferred to the hydroponic culture system. Plantlets were placed on a perforated opaque plate (4 holes per plate, 2 plants per hole, held in place by a foam plug) (Supplementary Fig. S1). The plate was then placed on the top of a sterile borosilicate glass beaker (diameter 80 mm, height 150 mm, no spout) filled with 640 mL of nutrient solution within 1 cm of the top of the beaker (Plant-Prod^®^ N: K:P, 14:12:32 commercialized by Fertil supplemented with the Hortrilon^®^ microelements from Compo, diluted at 1/2000 in ultrapure milli-Q water from a Milli-Q^®^ Advantage A10 water purification system). The entire root system was in contact with the culture medium. The beaker was placed in an opaque plastic pot to protect the roots from light. Eight plants of each variety were grown per beaker and 2 beakers of each variety were prepared for each experiment. Four biological replicates were conducted. The plants were grown in a cabinet with the same growth conditions as before. Beakers and nutrient solution were changed every 2 days to reduce contamination.

### Root exudate collection

After 10 days in hydroponics, the plants were removed from the beakers, the roots were rinsed in 650 mL of milli-Q water and placed on the top of a sterile borosilicate glass beaker (diameter 60 mm, height 120 mm, no spout) filled with 260 mL of milli-Q water and the roots were kept in the dark. Exudates were collected 24 h after root immersion. The volume of exudates was measured using a glass graduated cylinder and centrifuged at 3,166 x g (g-force or gravitational force equivalent) for 20 min to remove root debris. Supernatants were aliquoted into sterile 50 mL Falcon tubes (40 mL per tube) and stored at -20 °C prior to lyophilisation. Water was preferred to the nutrient solution for exudation to avoid the presence of excessive amounts of salt in exudates, which were shown to interfere with chromatographic and enzymatic analyses (data not shown). This analysis was conducted for each variety with 2 beakers per experiment with 4 or 5 experimental replicates.

### Plant tissue collection

The entire aboveground (shoots) and root systems (roots) of the plants from each beaker were collected separately after exudation (the 8 shoots or roots of each beaker were pooled) for the various analysis. Samples were weighed fresh, quickly frozen in liquid nitrogen and stored at -80 °C prior to analysis. Dry weights were measured after lyophilisation.

### Total carbon analysis in roots and exudates

Carbon (C) content in roots and exudates was determined using an Elemental Analyzer. The C content was expressed per plant to reflect the C fixation capacity of a single plant or per mg of leaf dry weight to compare the two plant species with different growth capacities and architectures. One milligram of lyophilised roots was placed in tin capsules and incinerated in an elemental analyser (Pyrocube, Elementar^®^, Lyon, France). A total volume of 120 mL (3*40 mL) of each exudate was reconstituted in 1.5 mL of sterile milliQ water, then lyophilised and concentrated in 200 µL of sterile water. These 200 µL were placed in capsules and allowed to evaporate at 30 °C for 1 week. A quantity of ~ 0.6 mg of powder was then analysed using the elemental analyser. The resulting N_2_ and CO_2_ gases were separated on two gas selective columns coupled to a thermal conductivity detector (TCD). They were quantified against standards (ammonium sulphate 21.2% N, benzoic acid 68.85% C, glutamic acid 9.51% N / 40.82% C and glutamine 19.17% N / 41.09% C). The elemental C and nitrogen contents are given in % (mass fraction). The results are expressed in mg of C per plant or in mg C/mg leaf dry weight. For exudates, C content was reported to the total volume of exudation.

### Non-targeted metabolic profiling by GC-MS of exudates and roots

Volumes of 40 mL of exudates were lyophilized as described before. Exudates were reconstituted in 1 mL of a water/acetonitrile/isopropanol (2:3:3) solution, shaken at 20 °C, 10 min, 1,500 rpm and centrifuged at 15,300 x g for 10 min. Fifty µL of supernatant was mixed with 100 µL of the water/acetonitrile/isopropanol (2:3:3) solution containing 4 µg/mL of ribitol (as internal standard) and dried overnight in a SpeedVac evaporator at 30 °C.

The roots were crushed in liquid nitrogen and dried by lyophilisation. Metabolites were extracted from 50 mg of root powder by triple hydroalcoholic extraction with cold 80% ethanol. After centrifugation 10 min at 16,900x g, the three supernatants were combined and dried overnight in a SpeedVac evaporator at 30 °C. Samples were then reconstituted in 100 µL of Milli-Q sterile water. A volume of 150 µL of the water/acetonitrile/isopropanol (2:3:3) solution containing 4 µg/mL of ribitol (as internal standard) was added to 25 µL of the root samples and then dried overnight in a SpeedVac evaporator at 30 °C.

All steps for GC-MS analyses were carried out as described previously with the same protocol root and exudate samples^63,64^. Dried samples were resuspended in 150 µL of the extraction solution containing 4 µg/mL of ribitol (as internal standard) and dried a second time in a SpeedVac evaporator for 2 h at 30 °C before adding 10 µL of 20 mg/mL methoxyamine in pyridine to the samples and the first step of derivatization was performed for 90 min at 30 °C under continuous shaking in an Eppendorf thermomixer. Then 90 µL of N-methyl-N-trimethylsilyl- trifluoroacetamide (MSTFA) (Regis Technologies, Morton Grove, IL, USA) were added and the reaction continued for 30 min at 37 °C. After cooling, all samples were transferred to an Agilent vial for injection.

Four hours after derivatisation, 1 µL of each sample was injected into an Agilent 7890B gas chromatograph coupled to an Agilent 5977 A mass spectrometer. The column was a Restek Rxi-5SilMS (30 m with 10 m Integra-Guard column). A split mode injection with a ratio of 1:30 was systematically performed for the quantification of saturated compounds. The oven temperature ramp was 60 °C for 1 min then 10 °C/min to 325 °C for 10 min. The constant helium flow was 1.1 mL/min. Temperatures were as follows: injector 250 °C, transfer line 290 °C, source 230 °C and quadrupole 150 °C. The quadrupole mass spectrometer was switched on after a solvent delay time of 5.90 min and scanned from 50 to 600 m/z. Absolute retention times were locked to the internal standard ribitol using the RTL system provided in Agilent’s Masshunter software. The Agilent Fiehn GC/MS Metabolomics RTL Library (June 2008 version) was used for metabolite identification^46^. Peak areas were determined using Masshunter Quantitative Analysis (Agilent Technologies, Santa Clara, CA, USA) in splitless and split 30 modes. The resulting areas were compiled into a single MS Excel file for comparisons. Peak areas were normalised to ribitol, leaf dry weight and the total volume of exudates (for exudation samples). A total of 143 metabolites were identified and their relative abundance (semi-quantitative determination) expressed in arbitrary units (a.u. per mg of leaf DW). It should be noted that the Nacre root samples (vetch) are missing from the GC-MS metabolic analysis. The four oat root samples were analysed, as were the exudate samples from the four varieties of vetch and oats. The result of the GC-MS analysis for the 143 metabolites used in Figs. 4 and 5 are given in Supplementary Table 2.

### Enzymatic assay for soluble sugar quantification in roots

The enzymatic assay was done based on the protocol described by^65^. The roots were ground to powder in the presence of liquid nitrogen. Soluble sugars were extracted from 50 mg of root powder by triple hydroalcoholic extraction with cold 80% ethanol. After centrifugation (10 min at 16,900 x g), the three supernatants were combined and dried using a SpeedVac evaporator at 30 °C overnight. The dry pellets obtained were resuspended in 100 µL of sterile Milli-Q water. Sucrose and hexose contents were determined by using an enzymatic sugar kit (Sucrose/D-glucose/D-fructose kit, R-BioPharm, Germany), by measuring the conversion of NAD^+^ to NADPH at 340 nm with a nanospectrophotometer (TECAN). The concentration of soluble sugars was calculated using a sucrose standard curve and a glucose standard curve. The results were expressed as mg of sugars (sucrose, glucose and fructose)/mg of root dry weight.

### HPLC for soluble sugar quantification in exudates

A volume of 40 mL of each exudate was lyophilized to dryness. Exudates were reconstituted in 1 mL of Milli-Q water, then dried using a SpeedVac evaporator at 30 °C overnight, and reconstituted again in 100 µL of water (concentration factor of exudates ~ 400 X). Ten µL of the resulting solution was injected in the HPLC Agilent 1260 Infinity system. Sucrose, glucose and fructose were separated using a cation exchange column (SugarPack 10 μm, 6.5 × 300 mm, Waters^®^) at 90 °C, with isocratic milli-Q water as a mobile phase (flow rate 0.5 mL/min). Detection was performed using an Agilent 1260 Infinity refractive index detector. All samples were injected using the autosampler. The chromatography data were analysed by ChemStation software. Peak identity was confirmed by co-elution with authentic standards. Sugar contents were calculated with a calibration curve using the peak area of a compound of interest and taking into account the total volume of exudation. The results are expressed in mg of sugars (sucrose, glucose and fructose)/mg of leaf dry weight.

### Statistical analysis

R version 4.2.1 (R Core Team and RStudio) was used for statistical analyses. Data normality and variance homogeneity were assessed using Shapiro-Wilk and Bartlett tests. To compare vetch and oat varieties, one-way ANOVA with Tukey’s post-hoc tests were used when data was normally distributed and homoscedastic. When data were non-normally distributed, the Kruskal–Wallis test with Dunn’s post-hoc correction were used. To compare vetch and oat, Student t-test was used for normally distributed data, or Wilcoxon rank-sum were applied otherwise. For all tests α = 0.05 corresponds to a 95% confidence interval. Principal Component Analysis (PCA) was performed on the different metabolites identified and semi-quantified by GC-MS in order to study the variation between varieties and between vetch and oat.

## Supplementary Information

Below is the link to the electronic supplementary material.

Supplementary Material 1

Supplementary Material 2

## Data Availability

The authors confirm that the data supporting the findings of this study are available within the article [and/or] its supplementary materials. Supplementary information accompanies this paper at https://nnnnn**Supplementary Fig. S1** : Description of the hydroponic culture system for common vetch and bristle oat.**Supplementary Table S1** : Metabolite classes detected in roots, exudates and in both roots and exudates.**Supplementary Table S2:** GC MS analysis metabolites raw data.

## References

[1] Terrer, C. et al. A trade-off between plant and soil carbon storage under elevated CO_2_. *Nature* **591**, 599–603 (2021). 33762765 10.1038/s41586-021-03306-8

[2] Rasmussen, J., Gylfadóttir, T., Dhalama, N. R., De Notaris, C. & Kätterer, T. Temporal fate of 15 N and 14 C leaf-fed to red and white clover in pure stand or mixture with grass – Implications for estimation of legume derived N in soil and companion species. *Soil. Biol. Biochem.* **133**, 60–71 (2019).

[3] Villarino, S. H., Pinto, P., Jackson, R. B. & Piñeiro, G. Plant rhizodeposition: A key factor for soil organic matter formation in stable fractions. *Sci. Adv.* **7**, eabd3176 (2021). 33853771 10.1126/sciadv.abd3176 PMC8046368

[4] Pausch, J. & Kuzyakov, Y. Carbon input by roots into the soil: Quantification of rhizodeposition from root to ecosystem scale. *Glob Change Biol.* **24**, 1–12 (2018). 10.1111/gcb.13850 28752603

[5] Keller, A. B., Brzostek, E. R., Craig, M. E., Fisher, J. B. & Phillips, R. P. Root-derived inputs are major contributors to soil carbon in temperate forests, but vary by mycorrhizal type. *Ecol. Lett.* **24**, 626–635 (2021). 33492775 10.1111/ele.13651

[6] Sasse, J. Plant Chemistry and Morphological Considerations for Efficient Carbon Sequestration. *CHIMIA* **77**, 726–732 (2023). 38047839 10.2533/chimia.2023.726

[7] Liang, Z., Rasmussen, J., Poeplau, C. & Elsgaard, L. Priming effects decrease with the quantity of cover crop residues – Potential implications for soil carbon sequestration. *Soil. Biol. Biochem.* **184**, 109110 (2023).

[8] Lamichhane, J. R. & Alletto, L. Ecosystem services of cover crops: a research roadmap. *Trends Plant. Sci.* **27**, 758–768 (2022). 35459600 10.1016/j.tplants.2022.03.014

[9] Jacoby, R. P., Koprivova, A. & Kopriva, S. Pinpointing secondary metabolites that shape the composition and function of the plant microbiome. *J. Exp. Bot.* **72**, 57–69 (2021). 32995888 10.1093/jxb/eraa424 PMC7816845

[10] Garbeva, P., van Veen, J. A. & van Elsas, J. D. Microbial diversity in soil: Selection of Microbial Populations by Plant and Soil Type and Implications for Disease Suppressiveness. *Annu. Rev. Phytopathol.* **42**, 243–270 (2004). 15283667 10.1146/annurev.phyto.42.012604.135455

[11] Asghar, W. et al. Enhancing the Resilience of Agroecosystems Through Improved Rhizosphere Processes: A Strategic Review. *Int. J. Mol. Sci.* **26**, 109 (2024). 39795965 10.3390/ijms26010109 PMC11720004

[12] Çerçioğlu, M., Udawatta, R. P. & Anderson, S. H. Use of cover crops for sustainable management of soil condition and health: A review. *Soil. Secur.* **18**, 100177 (2025).

[13] He, Q. et al. Optimizing cover cropping application for sustainable crop production. *Npj Sustain. Agric.* **3**, 10 (2025).

[14] Launay, C. et al. Estimating the carbon storage potential and greenhouse gas emissions of French arable cropland using high-resolution modeling. *Glob Change Biol.* **27**, 1645–1661 (2021). 10.1111/gcb.15512 33421219

[15] Zhou, X., Wu, H., Li, G. & Chen, C. Short-term contributions of cover crop surface residue return to soil carbon and nitrogen contents in temperate Australia. *Environ. Sci. Pollut Res.* **23**, 23175–23183 (2016). 10.1007/s11356-016-7549-5 27600727

[16] Poeplau, C. & Don, A. Carbon sequestration in agricultural soils via cultivation of cover crops – A meta-analysis. *Agric. Ecosyst. Environ.* **200**, 33–41 (2015).

[17] Streit, J., Meinen, C., Nelson, W. C. D., Siebrecht-Schöll, D. J. & Rauber, R. Above- and belowground biomass in a mixed cropping system with eight novel winter faba bean genotypes and winter wheat using FTIR spectroscopy for root species discrimination. *Plant. Soil.* **436**, 141–158 (2019).

[18] Xiao, X. et al. Intercropping enhances microbial community diversity and ecosystem functioning in maize fields. *Front. Microbiol.* **13**, 1084452 (2023). 36687629 10.3389/fmicb.2022.1084452 PMC9846038

[19] Vranova, V., Rejsek, K., Skene, K. R., Janous, D. & Formanek, P. Methods of collection of plant root exudates in relation to plant metabolism and purpose: A review. *J. Plant. Nutr. Soil. Sci.* **176**, 175–199 (2013).

[20] Mortensen, E. Ø., De Notaris, C., Peixoto, L., Olesen, J. E. & Rasmussen, J. Short-term cover crop carbon inputs to soil as affected by long-term cropping system management and soil fertility. *Agric. Ecosyst. Environ.* **311**, 107339 (2021).

[21] McClelland, S. C., Paustian, K. & Schipanski, M. E. Management of cover crops in temperate climates influences soil organic carbon stocks: a meta-analysis. *Ecol. Appl.* **31**, e02278 (2021). 33320994 10.1002/eap.2278

[22] Poeplau, C. et al. Cover crops do increase soil organic carbon stocks—A critical comment on Chaplot and Smith (2023). *Glob. Change Biol.* **30**, e17128 (2024). 10.1111/gcb.17128 38273485

[23] Restovich, S. B., Andriulo, A. E. & Portela, S. I. Cover crop mixtures increase ecosystem multifunctionality in summer crop rotations with low N fertilization. *Agron. Sustain. Dev.* **42**, 19 (2022).

[24] Guyonnet, J. P. et al. The effects of plant nutritional strategy on soil microbial denitrification activity through rhizosphere primary metabolites. *FEMS Microbiol. Ecol.* **93**, (2017). 10.1093/femsec/fix022 28334144

[25] Voges, M. J. E. E. E., Bai, Y., Schulze-Lefert, P. & Sattely, E. S. Plant-derived coumarins shape the composition of an *Arabidopsis* synthetic root microbiome. *Proc. Natl. Acad. Sci.* **116**, 12558–12565 (2019). 10.1073/pnas.1820691116 PMC6589675 31152139

[26] Cazzaniga, S. G. et al. On the legacy of cover crop-specific microbial footprints. *Soil. Biol. Biochem.* **184**, 109080 (2023).

[27] Sokol, N. W., Kuebbing, S. E., Karlsen-Ayala, E. & Bradford, M. A. Evidence for the primacy of living root inputs, not root or shoot litter, in forming soil organic carbon. *New. Phytol*. **221**, 233–246 (2019). 30067293 10.1111/nph.15361

[28] Badri, D. V. & Vivanco, J. M. Regulation and function of root exudates. *Plant. Cell. Environ.* **32**, 666–681 (2009). 19143988 10.1111/j.1365-3040.2008.01926.x

[29] Bais, H. P., Weir, T. L., Perry, L. G., Gilroy, S. & Vivanco, J. M. The role of root exudates in rhizosphere interactions with plants and other organisms. *Annu. Rev. Plant. Biol.* **57**, 233–266 (2006). 16669762 10.1146/annurev.arplant.57.032905.105159

[30] Rasmann, S. & Turlings, T. C. Root signals that mediate mutualistic interactions in the rhizosphere. *Curr. Opin. Plant. Biol.* **32**, 62–68 (2016). 27393937 10.1016/j.pbi.2016.06.017

[31] Bouwmeester, H., Dong, L., Wippel, K., Hofland, T. & Smilde, A. The chemical interaction between plants and the rhizosphere microbiome. *Trends Plant. Sci.* **30**, 1002–1019 (2025). 40603217 10.1016/j.tplants.2025.06.001 PMC12415530

[32] Vives-Peris, V., de Ollas, C., Gómez-Cadenas, A. & Pérez-Clemente, R. M. Root exudates: from plant to rhizosphere and beyond. *Plant. Cell. Rep.* **39**, 3–17 (2020). 31346716 10.1007/s00299-019-02447-5

[33] Chen, L. & Liu, Y. The Function of Root Exudates in the Root Colonization by Beneficial Soil Rhizobacteria. *Biology* **13**, 95 (2024). 38392313 10.3390/biology13020095 PMC10886372

[34] Herz, K. et al. Linking root exudates to functional plant traits. *PLOS ONE*. **13**, e0204128 (2018). 30281675 10.1371/journal.pone.0204128 PMC6169879

[35] Heuermann, D. et al. Distinct metabolite classes in root exudates are indicative for field- or hydroponically-grown cover crops. *Front. Plant. Sci.* **14**, 1122285 (2023). 37089658 10.3389/fpls.2023.1122285 PMC10118039

[36] Domeignoz-Horta, L. A. et al. Plant diversity drives positive microbial associations in the rhizosphere enhancing carbon use efficiency in agricultural soils. *Nat. Commun.* **15**, 8065 (2024). 39277633 10.1038/s41467-024-52449-5 PMC11401882

[37] Qiao, M. et al. Legume rhizodeposition promotes nitrogen fixation by soil microbiota under crop diversification. *Nat. Commun.* **15**, 2924 (2024). 38575565 10.1038/s41467-024-47159-x PMC10995168

[38] Frasier, I. et al. High quality residues from cover crops favor changes in microbial community and enhance C and N sequestration. *Glob Ecol. Conserv.* **6**, 242–256 (2016).

[39] McLaughlin, S., Zhalnina, K., Kosina, S., Northen, T. R. & Sasse, J. The core metabolome and root exudation dynamics of three phylogenetically distinct plant species. *Nat. Commun.* **14**, 1649 (2023). 36964135 10.1038/s41467-023-37164-x PMC10039077

[40] Petkova, M., Shilev, S., Popova, V., Neykova, I. & Minev, N. Intercropping of Oats with Vetch Conducts to Improve Soil Bacteriome Diversity and Structure. *Microorganisms* **13**, 977 (2025). 40431149 10.3390/microorganisms13050977 PMC12114406

[41] Liu, X. et al. Root Architecture of Forage Species Varies with Intercropping Combinations. *Agronomy* **13**, 2223 (2023).

[42] Hudek, C., Putinica, C., Otten, W. & De Baets, S. Functional root trait-based classification of cover crops to improve soil physical properties. *Eur. J. Soil. Sci.* **73**, e13147 (2022).

[43] Concha, C. & Doerner, P. The impact of the rhizobia–legume symbiosis on host root system architecture. *J. Exp. Bot.* **71**, 3902–3921 (2020). 32337556 10.1093/jxb/eraa198 PMC7316968

[44] Joseph Fernando, E. A. et al. Going deep: Roots, carbon, and analyzing subsoil carbon dynamics. *Mol. Plant.* **17**, 1–3 (2024). 38008936 10.1016/j.molp.2023.11.009

[45] Mortensen, E. Ø. et al. Smart Mixture Design Can Steer the Fate of Root-Derived Carbon Into Mineral‐Associated and Particulate Organic Matter in Intensively Managed Grasslands. *Glob Change Biol.* **31**, e70117 (2025). 10.1111/gcb.70117 PMC11883481 40045867

[46] Salem, M. A., Wang, J. Y. & Al-Babili, S. Metabolomics of plant root exudates: From sample preparation to data analysis. *Front. Plant. Sci.* **13**, 1062982 (2022). 36561464 10.3389/fpls.2022.1062982 PMC9763704

[47] Gargallo-Garriga, A. et al. Root exudate metabolomes change under drought and show limited capacity for recovery. *Sci. Rep.* **8**, 12696 (2018). 30140025 10.1038/s41598-018-30150-0 PMC6107494

[48] Boutet, S. et al. Untargeted metabolomic analyses reveal the diversity and plasticity of the specialized metabolome in seeds of different *Camelina sativa* genotypes. *Plant. J.* **110**, 147–165 (2022). 34997644 10.1111/tpj.15662

[49] Fracchia, F. et al. Microbial colonisation rewires the composition and content of poplar root exudates, root and shoot metabolomes. *Microbiome* **12**, 173 (2024). 39267187 10.1186/s40168-024-01888-9 PMC11395995

[50] Couvillion, S. P. et al. Root exudate lipids: Uncovering chemodiversity and carbon stability potential. *Soil. Biol. Biochem.* **206**, 109799 (2025).

[51] Bag, S., Mondal, A., Majumder, A., Mondal, S. K. & Banik, A. Flavonoid mediated selective cross-talk between plants and beneficial soil microbiome. *Phytochem Rev.* **21**, 1739–1760 (2022). 35221830 10.1007/s11101-022-09806-3 PMC8860142

[52] Liu, H. et al. Nucleotides enriched under heat stress recruit beneficial rhizomicrobes to protect plants from heat and root-rot stresses. *Microbiome* **13**, 160 (2025). 40624576 10.1186/s40168-025-02126-6 PMC12235776

[53] Pantigoso, H. A., Manter, D. K., Fonte, S. J. & Vivanco, J. M. Root exudate-derived compounds stimulate the phosphorus solubilizing ability of bacteria. *Sci. Rep.* **13**, 4050 (2023). 36899103 10.1038/s41598-023-30915-2 PMC10006420

[54] He, D. et al. Flavonoid-attracted *Aeromonas* sp. from the Arabidopsis root microbiome enhances plant dehydration resistance. *ISME J.* **16**, 2622–2632 (2022). 35842464 10.1038/s41396-022-01288-7 PMC9561528

[55] Wu, J. et al. Flavones enrich rhizosphere Pseudomonas to enhance nitrogen utilization and secondary root growth in Populus. *Nat. Commun.* **16**, 1461 (2025). 39920117 10.1038/s41467-025-56226-w PMC11805958

[56] Subrahmaniam, H. J. et al. Natural variation in root exudate composition in the genetically structured *Arabidopsis thaliana* in the Iberian Peninsula. *New. Phytol*. **245**, 1437–1449 (2025). 39658885 10.1111/nph.20314 PMC11754937

[57] Oburger, E. & Jones, D. L. Sampling root exudates –. *Mission impossible? Rhizosphere*. **6**, 116–133 (2018).

[58] Aulakh, M. S., Wassmann, R., Bueno, C., Kreuzwieser, J. & Rennenberg, H. Characterization of Root Exudates at Different Growth Stages of Ten Rice (*Oryza sativa* L.) Cultivars. *Plant. Biol.* **3**, 139–148 (2001).

[59] Ayers, W. A. & Thornton, R. H. Exudation of amino acids by intact and damaged roots of wheat and peas. *Plant. Soil.* **28**, 193–207 (1968).

[60] Carvalhais, L. C. et al. Root exudation of sugars, amino acids, and organic acids by maize as affected by nitrogen, phosphorus, potassium, and iron deficiency. *J. Plant. Nutr. Soil. Sci.* **174**, 3–11 (2011).

[61] Phillips, D. A., Fox, T. C., King, M. D., Bhuvaneswari, T. V. & Teuber, L. R. Microbial Products Trigger Amino Acid Exudation from Plant Roots. *Plant. Physiol.* **136**, 2887–2894 (2004). 15347793 10.1104/pp.104.044222 PMC523350

[62] Ehrhardt, D. W., Atkinson, E. M. & Long, S. R. Depolarization of Alfalfa Root Hair Membrane Potential by *Rhizobium meliloti* Nod Factors. *Science* **256**, 998–1000 (1992). 10744524 10.1126/science.10744524

[63] Fiehn, O. Metabolite Profiling in *Arabidopsis*. in Arabidopsis Protocols vol. 323 439–448 (Humana, New Jersey, (2006). 10.1385/1-59745-003-0:439 16739598

[64] Fiehn, O. et al. Quality control for plant metabolomics: reporting MSI-compliant studies. *Plant. J.* **53**, 691–704 (2008). 18269577 10.1111/j.1365-313X.2007.03387.x

[65] Sellami, S. et al. Salinity Effects on Sugar Homeostasis and Vascular Anatomy in the Stem of the Arabidopsis Thaliana Inflorescence. *Int. J. Mol. Sci.* **20**, 3167 (2019). 31261714 10.3390/ijms20133167 PMC6651052

