# Supplementary material for: Exudate compositions differ between the cover crops vetch and oat
Source: Sci Rep. 2026 Mar 22;16:14517. doi: 10.1038/s41598-026-44751-7 (PMC13149867; doi:10.1038/s41598-026-44751-7)
Supplement: Supplementary file 1 — Supplementary Material 1 [file 41598_2026_44751_MOESM1_ESM.docx]

**Supplementary information**


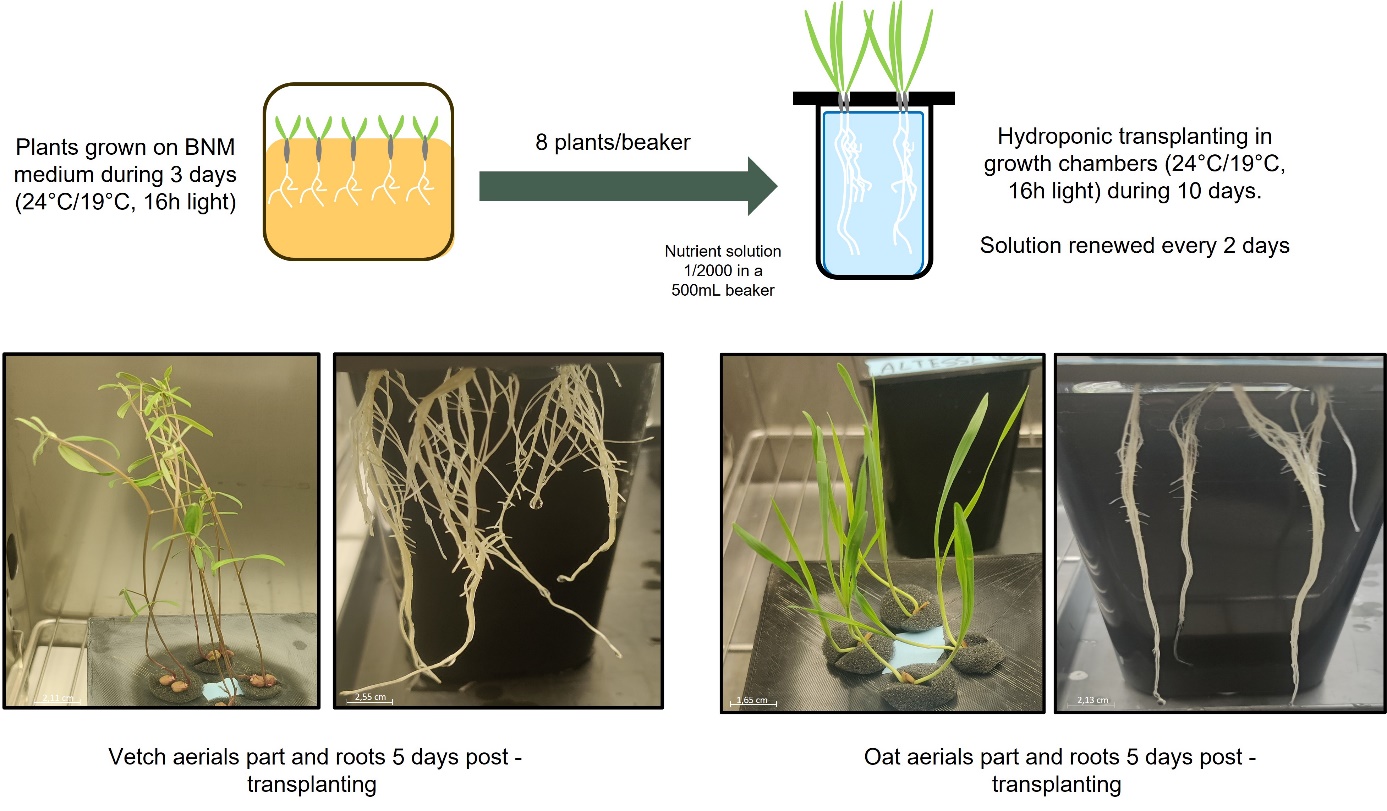


**Supplementary Figure S1: Description of the hydroponic culture system for common vetch and bristle oat.** The upper part of the figure describes some steps of hydroponics establishment: After surface-sterilisation, stratification at 4°C and germination for 24 h in the dark (see the ‘Methods’ section), plantlets were grown for 3 days on BNM agar plates (with roots sandwiched between two pieces of sterile paper). Plantlets of homogeneous size were then transferred to the hydroponic culture system. Plantlets were placed on a perforated opaque plate (4 holes per plate, 2 plants per hole, plants held in place by a foam plug). The plate was then placed on the top of a sterile borosilicate glass beaker filled with the nutrient solution diluted at 1/2000. The beaker was placed in an opaque plastic pot to protect the roots from light. Beakers and nutrient solution were changed every 2 days to reduce contamination. The lower part of the figure shows the aerial parts and root systems of vetch and oat 5 days after the beginning of hydroponic culture.


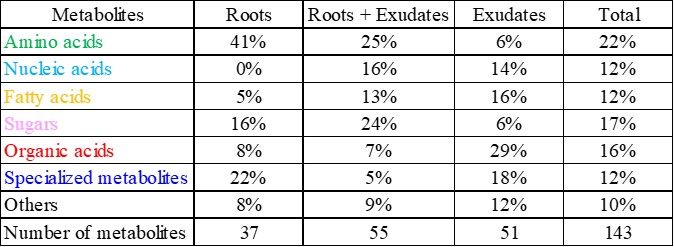


**Supplementary Table S1:** **Metabolite classes detected in roots, exudates and in both roots and exudates.** For each class of metabolites, percentages of specific metabolites present in roots, exudates or in both roots and exudates are indicated. The last column shows the distribution of total metabolites according to the different classes of metabolites. The last line indicates the total number of specific metabolites in each compartment.
